# miR-21 regulates immunosuppression mediated by myeloid-derived suppressor cells by impairing RUNX1-YAP interaction in lung cancer

**DOI:** 10.1186/s12935-020-01555-7

**Published:** 2020-10-12

**Authors:** Guangping Meng, Jinying Wei, Yanjun Wang, Danhua Qu, Jie Zhang

**Affiliations:** 1 Department of Respiratory and Critical Care Medicine, The Second Hospital of Jilin University, No. 218, Ziqiang Street, Changchun, 130000 Jilin People’s Republic of China; 2 Department of General Practice, The First Hospital of Jilin University, Changchun, 130021 People’s Republic of China; 3 Department of Nursing, The Second Hospital of Jilin University, Changchun, 130000 People’s Republic of China

**Keywords:** Lung cancer, microRNA-21, Runt-related transcription factor 1, Yes-associated Protein, Myeloid-derived suppressor cells, Immunosuppressive ability

## Abstract

**Background:**

Myeloid-derived suppressor cells (MDSCs) are known suppressors of antitumor immunity and contribute to immunosuppressive microenvironment during tumor development including lung cancer. Accumulating evidence shows microRNAs (miRNAs) affect tumor-expanded MDSC accumulation and function in tumor microenvironment and favor solid tumor growth. Herein, we aim to characterize the role of miR-21 in regulating the accumulation and activity of MDSCs in lung cancer.

**Methods:**

The proportions of MDSCs, T helper cells (Th), and cytotoxic T lymphocytes (CTL) were evaluated by flow cytometric analyses of peripheral blood and tumor tissues collected from Lewis lung-cancer-bearing mice. T cell proliferation assay was performed in CD4+ or CD8+ T cells cocultured with MDSCs. MDSC apoptosis was examined by flow cytometric analysis. The levels of IL-10, TGF-β, and GM-CSF in mouse serum were determined by ELISA. miR-21 targeting RUNX1 and RUNX1 interaction with YAP were evaluated by RIP, dual-luciferase reporter gene, and ChIP assays.

**Results:**

MiR-21 inhibition by its antagomir reduced the proportion of MDSCs, increased the proportion of Th and CTL in peripheral blood and tumor tissues of Lewis lung-cancer-bearing mice, protected Th and CTL from the suppression of MDSCs, increased apoptosis of MDSCs, but reduced IL-10, TGF-β and GM-CSF levels in mouse serum. RUNX1 could transcriptionally inhibit the YAP expression, whereas miR-21 targeting RUNX1 led to elevated YAP expression levels. Mechanistic investigation showed that miR-21 maintained MDSC accumulation in tumor microenvironment and promoted immunosuppressive ability of MDSCs in Lewis lung-cancer-bearing mice by down-regulating RUNX1and up-regulating YAP.

**Conclusions:**

Taken together, the study provides evidence that targeting miR-21 in MDSCs may be developed as an immunotherapeutic approach to combat lung cancer development.

## Background

Lung cancer is one of the widely-diagnosed cancers around the world, and remains one of the leading causes of cancer-associated mortality in both men and women [1]. Moreover, less than 7% of patients survive 10 years after diagnosis across all stages of lung cancer [2]. Metastasis to other organs, especially the brain, is considered to be responsible for the high mortality rate associated with lung cancer [3]. Nowadays, the implementation of immunotherapy has become more and more encouraging and promising in the treatment and management of lung cancer [4]. Meanwhile, myeloid-derived suppressor cells (MDSCs), a heterogeneous group of immune cells from the myeloid lineage, which serve as suppressors of antitumor immunity, have been found to contribute to the immunosuppressive microenvironment during tumor development [5]. Therapeutic strategies targeting MDSCs in combination with primary mammary tumor resection has also been reported to reduce and retard metastatic growth in the lungs in mice bearing orthotopic murine mammary tumors [6]. Additionally, studies have shown that targeting MDSC recruitment and enhancing antitumor immunity can augment the therapeutic efficacy of ablative hypofractionated radiation therapy in subcutaneous tumors using syngeneic lung cancer [7]. Considering the emerging role of MDSCs in lung cancer, improved therapeutic regimens targeting MDSCs could prove useful in controlling the development and progression of lung cancer. Increasing evidence has further shown that microRNAs (miRNAs), such as miR-494 and miR-155, influence tumor-expanded MDSC accumulation, and also function in tumor microenvironment and favor solid tumor growth [8, 9].

MiRNAs are a group of endogenous RNAs of about 21 nucleotides in length and play numerous regulatory roles in animals and plants [10]. The novel ability of miRNAs to regulate the expression of oncogenic pathways and their vital roles in lung cancer progression indicate that miRNAs could potentially serve as prognostic biomarkers or targets for treatment of cancers [11]. Moreover, studies have demonstrated that over-expression of miR-21-5p by mesenchymal stem cell-secreted extracellular vesicles (MSC-EVs) promotes the development of lung cancer [12]. In addition, suppressing miR-21 inhibited cell migration and invasion in non-small cell lung carcinoma (NSCLC) cells [13]. Bioinformatics analysis revealed the RUNX1 transcription factor as one of the downstream targets of miR-21. The runt-related transcription factor (RUNX) family (RUNX1, RUNX2, and RUNX3), which is involved in many cell lineages, has been widely-associated with the development of human cancers, such as acute myeloid leukemia (PMID: 21447743), colon carcinoma (PMID: 22396198), and hepatocellular carcinoma (PMID: 21328447) [14, 15]. Even more so, RUNX1, a core-binding factor in transcription families, is one of the most commonly mutated genes found in various hematological malignancies [16]. A prior study also revealed shown that RUNX1 can inhibit Yes-associated protein (YAP) to accelerate the occurrence of tumor [17]. Therapeutic activation of YAP, a Hippo pathway effector, is known to bring about severe side effects in human cancer development [18, 19]. Besides, miR-129 has also been found to directly-suppress the expression of RUNX1 and mediate the transcriptional modulation by RUNX1 [20]. In this regard, we hypothesized that a regulatory network of the miR-21/RUNX1/YAP axis may be implicated in the progression of lung cancer. Therefore, the current study was conducted with the aim to verify the expected involvement of the miR-21/RUNX1/YAP axis in lung cancer, and to elucidate the underlying molecular mechanisms.

## Materials and methods

### Ethics statement

All animal experimentation and procedures were approved by the Institutional Animal Care and Use Committee of the Second Hospital of Jilin University. The animal experiments were conducted based on minimized animal numbers and discomfort of experimental animals.

### Microarray-based analysis

Firstly, the lung cancer related miRNA dataset GSE63805 was retrieved from the Gene Expression Omnibus (GEO) database (https://www.ncbi.nlm.nih.gov/gds), which comprised of 62 tissue samples, including 30 normal lung samples and 32 lung cancer samples. Differential analysis of the expression datasets was subsequently performed using the limma package (http://www.bioconductor.org/packages/release/bioc/html/limma.html) of the R language, with the threshold (|logFC| > 1, *p *< 0.01). The miR-21 exhibiting the lowest *p* value was selected for further experimentation. The downstream target genes of the miRNA were predicted with the help of mirDIP (Integrated Score > 0.2) (http://ophid.utoronto.ca/mirDIP/) and starbase (clipExpNum ≥ 3) (http://starbase.sysu.edu.cn). In addition, differential expression analysis was also performed on the lung cancer dataset GSE74706 with the R language (|logFC| > 1, *p *< 0.01), which comprised of 36 tissue samples, including 18 normal lung samples and 18 lung cancer samples. Then, the human transcription factors were obtained from the Cistrome database (http://cistrome.org), and these results were intersected to determine the downstream target gene of miR-21 based on existing data. Next, the related genes of the target gene were identified using the GeneMANIA database (http://genemania.org), and Cistrome was again employed to obtain the genes with a correlation greater than 0.35 or less than -0.35 with the expression of downstream genes of target transcription factors in lung adenocarcinoma. Results of GeneMANIA and Cistrome results were then intersected to obtain the downstream genes of the key target transcription factors. Moreover, the comprehensive data of lung adenocarcinoma and squamous cell carcinoma in the Cancer Genome Atlas (TCGA) database (http://gepia.cancer-pku.cn) was analyzed using the Gene Expression Profiling Interactive Analysis (GEPIA) database (http://gepia.cancer-pku.cn) to confirm the correlation of expressions between the target transcription factors and their downstream genes, and starBase was used to determine the binding site of miRNA and target transcription factors.

### Cell culture and treatment

Lewis lung cancer cells were purchased from Cancer Research Institute of Chinese Academy of Medical Sciences (Beijing, China). The obtained cells were subsequently cultured in Dulbecco’s Modified Eagle Medium (DMEM) containing 10% fetal bovine serum (TIANHANG, Hangzhou, China), 100 U/mL penicillin and 100 U/mL streptomycin at 37 °C in a humidified atmosphere with 5% CO_2_. The cells were observed daily as they grew into a monolayer adhering to the chamber walls. Upon reaching the logarithmic phase of growth, the cells were trypsinized and passaged with 0.25% trypsin solution once every 2–3 days. Trypan blue staining was then employed to assess the ratio of living cells. Later, the cell concentration was diluted to 1 × 10^7^ cells/mL with phosphate buffer saline (PBS), and the cell suspension was prepared for further experimentation.

### Animal model establishment

A total of 72 C57BL/6 mice (36 males & 36 females, aged 5–6 weeks) were purchased from Beijing Vital River Laboratory Animal Technology Co., Ltd., Beijing, China) for animal experimentation in the current study. The mice meeting the average weight of (20 ± 0.5) g were numbered after acclimation for 1 week, and then 8 mice (4 males and 4 females) were randomly selected as the blank control, and subcutaneously injected with 0.4 mL normal saline into the armpit of the right forelimb. The remaining 64 mice were simultaneously inoculated with 0.2 mL Lewis lung cancer cell suspension (about 2 × 10^6^ tumor cells). The tumor growth was observed and measured 3 days later.

When the tumor diameter had reached approximately 5 mm (around the 5th day), the 64 mice were infected with lentiviral particles containing miR-21 antagomir negative control (NC), miR-21 antagomir, miR-21 antagomir NC combined with sh-NC, miR-21 antagomir combined with sh-NC, miR-21 antagomir combined with sh-RUNX1, miR-21 antagomir NC combined with oe-NC, miR-21 antagomir combined with oe-NC, or miR-21 antagomir combined with oe-YAP, respectively, with 8 mice per inoculation. In brief, mice were intraperitoneally injected with 4 mg/kg normal saline (antagomir NC) or the same amount of miR-21 antagomir, while the other mice were also intraperitoneally injected with the same amounts of lentiviral particles or normal saline for a total of 5 times, respectively. Later, on the 7th, 14th and 21st day after inoculation, blood samples were collected from each mouse, and the tumor diameter was measured and recorded.

### Preparation of mouse tumor and peripheral blood cell suspension

After about 3 weeks of subcutaneous injection of Lewis lung cancer cells, the mice were intraperitoneally anesthetized with 3% pentobarbital sodium at a dose of 50 mg/kg. Peripheral blood samples obtained from the orbit were placed in 15 mL centrifuge tubes (containing 1 mL anticoagulant). The mice were then euthanized with cervical dislocation and the tumors were excised using scissors and forceps, before the tumors were placed in a 24-well plate with l mL PBS.

The tumor suspension was prepared as follows. In brief, the resected tumor was photographed, its dimensions (length and width) were measured with a Vernier caliper, and the tumor volume was calculated. The tumor was cut into small portions with scissors in a 24 hole plate, transferred to a 15 mL centrifuge tube, added with 2 mL 1640 culture solution and 60 μL collagenase, and incubated in a shaker at 37 °C for 3 h. After that, the centrifuge tube was mixed by vortexing, and added with PBS to a constant volume of 10 mL. After centrifugation, the supernatant was discarded and 2 mL PBS was added to the tube. Next, the solution was passed through a sieve into a 15 mL centrifuge tube, re-centrifuged with the supernatant discarded. Finally, PBS was added to establish the tumor cell suspension.

The peripheral blood cell suspension was prepared. In brief, 1 mL PBS was added to the 15 mL centrifuge tube containing anticoagulant, centrifuged and the supernatant was discarded. Upon the addition of 3 mL of hemolytic solution, the tube was placed aside for 4–5 min, and added with PBS to obtain the peripheral blood cell suspension.

### Flow cytometry for cell characterization

MDSCs were characterized as follows. A total of 50 µL of peripheral blood or tumor local cell suspension was transferred respectively into 2 mL centrifuge tubes, and 0.5 µL of CD45.2, CD11b and Gr-1 (BD Biosciences, Franklin Lakes, NJ, USA) was added to each tube. Next, the tubes were added with 48 µL of PBS, and stained in a refrigerator at 4 °C for 20 min. Then, the tubes were added with 1 mL of PBS and centrifuged at 300 rpm for 5 min, added with 100 µL PBS for resuspension, and finally analyzed using a flow cytometer.

T cell phenotype was detected. Briefly, 50 µL of peripheral blood and tumor local cell suspension was placed into 2 mL centrifuge tubes, and then mixed with 0.5 µL of CD45.2, CD4 and CD8a (BD Biosciences, Franklin Lakes, NJ, USA). Next, 48 µL of PBS was added to the tubes for staining in the refrigerator at 4 °C for 20 min. Then, the tubes were added with another 1 mL of PBS and centrifuged at 300 rpm. After removing the supernatant, 100 µL PBS was added for resuspension and analyzed with a flow cytometer.

### Flow cytometry for cell proliferation

Lymphocyte isolating medium was adopted to separate the cells in tumor tissues and peripheral blood samples. Next, the CD4^+^ or CD8^+^ T cells in the samples were separated using CD4^+^ or CD8^+^ T cell separation kits (Miltenyi Biotec, Bergisch Gladbach, Germany). According to the manufacturer’s instructions (Invitrogen Inc., Carlsbad, CA, USA), CD4^+^ or CD8^+^ T cells were labeled with 5-(and 6)-carboxyfluorescein diacetate, succinimidylester (CFSE), and then stimulated with Con A (Sigma-Aldrich Chemical Company, St Louis MO, USA). MDSCs and T cells were derived from mice infected with lentiviral particles or control mice in the co-culture experiments. For a single co-culture, the T cells and the MDSCs were derived from the same mice. Next, the T cells were co-cultured with MDSCs at the proportions of 2:1, 4:1, 10:1 or 100:1 in 96 well plates for 96 h. On the 4th day, the cells were analyzed with a flow cytometer. The proliferation data from MDSCs were obtained through the gradient experiments on average.

### MDSC sorting of peripheral blood

The mice were euthanized, and the peripheral blood samples were collected, followed by removal of red blood cells with a red blood cell lysis buffer. Next, the lysed blood was incubated at 4 °C for 15 min with the addition of biotin-conjugated anti-Gr-1 (BD Biosciences, Franklin Lakes, NJ, USA), and then added with anti-biotin beads and incubated in the dark at 4 °C for 15 min. After washing, the blood was resuspended with PBS, and MDSC sorting was performed using a LS column from Miltenyi Biotec (Bergisch Gladbach, Germany), following the manufacturer’s instructions.

### Flow cytometry for cell cycle analysis

MDSCs were collected and the cell concentration was adjusted to 1 × 10^6^ cells/mL. Next, the cells were seeded in 24 well plates, and treated with antagomir NC + sh-NC, miR-21 antagomir + sh-NC, miR-21 antagomir + sh-RUNX1, antagomir NC + oe-NC, miR-21 antagomir + oe-NC, or miR-21 antagomir + oe-YAP. After 24 h, the cells were collected, centrifuged at 2000 rpm for 5 min, fixed with 70% ethanol precooled at 4 °C, and stored at 4 °C. Prior to staining, the fixed solution was rinsed off with PBS, and the cells were resuspended with the addition of 200 µL PBS. A total of 100 µL RNase A was added to the cells in the water bath at 37 °C for 30 min, whereupon 400 µL propidium iodide (PI) was added. The mixture was then dyed at 4 ℃ for 30 min in conditions void of light. Before analysis, the cells were screened through a 200-mesh cell sieve, and added with 300 µL PBS to adjust the cell density. Then the cell cycle was analyzed using a flow cytometer, with the red fluorescence recorded at 488 nm, and 10000 cells were counted.

### Annexin V-FITC/PI analysis

MDSCs were collected and the cell density was adjusted to 1 × 10^6^ cells/mL. Next, the cells were seeded in 24 well plates, and then infected with antagomir NC + sh-NC, miR-21 antagomir + sh-NC, miR-21 antagomir + sh-RUNX1, antagomir NC + oe-NC, miR-21 antagomir + oe-NC, or miR-21 antagomir + oe-YAP. After 24 h, the cells were centrifuged at 2000 rpm for 5 min, rinsed twice with PBS, and suspended following the addition of 100 µL of binding buffer. Annexin V-FITC kits were employed for staining purposes. A total of 5 μL Annexin V-FITC was added to the cell suspension, and then 5 µL PI was added and allowed to react for 15 min in conditions void of light. Next, the cells were passed through a 200 mesh cell sieve. Finally, the cells were analyzed using a flow cytometer with an excitation wavelength of 488 nm and an emission wavelength of 530 nm, and 10000 cells were counted.

### Enzyme linked immunosorbent assay (ELISA)

The eyeballs of mice were removed to collect orbital blood samples, which were stored overnight at 4 °C, and then centrifuged at 3500×*g*. The clear serum from the upper layer was stored at − 80 °C. The serum levels of interleukin 10 (IL-10), transforming growth factor-beta (TGF-β) and granulocyte–macrophage colony stimulating factor (GM-CSF) were measured using ELISA kits. The serum was cultured for 24 h, whereupon the culture medium was collected, centrifuged at room temperature at 1000×*g* for 10 min, and the supernatant was collected. The standard curve was drawn and the contents of IL-10, TGF-β and GM-CSF in the cell culture medium were measured in strict accordance with the instructions of ELISA kit. All the aforementioned kits were purchased from Wuhan Xinqidi Biological Technology Co. Ltd. (Wuhan, China).

### RNA immunoprecipitation (RIP) assay

Lewis lung cancer cells were lysed with radio-immunoprecipitation assay (RIPA) cell lysis buffer (P0013B, Beyotime Biotechnology Co., Shanghai, China) on an ice bath for 5 min, and centrifuged at 12,000×*g* and 4 °C for 10 min. One portion of the cell extract was used as the input, while the remaining portion was incubated with antibody for co-precipitation. Each co-precipitation reaction system was rinsed with 50 μL magnetic beads and resuspended in 100 μL RIP wash buffer, and then incubated with 5 μg antibody for binding. After washing, the magnetic beads-antibody complex was resuspended in 900 μL RIP wash buffer and incubated overnight with 100 μL cell supernatant at 4 °C. The samples were then placed on magnetic pedestals to collect the beads-protein complexes, whereupon the samples and input were detached with treatment with protease K to extract RNA content for subsequent polymerase chain reaction (PCR) analysis. The antibodies used in the experiment were anti-RUNX1 (ab92336, Abcam Inc., Cambridge, UK) and immunoglobulin G (IgG, ab150077, Abcam Inc., Cambridge, UK), which served as NC.

### Chromatin immunoprecipitation (ChIP) assay

The Lewis lung cancer cells were fixed with formaldehyde for 10 min to induce DNA–protein cross-linking. Next, an ultrasonicator was used to break the chromatin into fragments for 15 cycles of 10 s each, with intervals of 10 sec. After that, the supernatant was collected, divided into two equal portions, and centrifuged at 12,000×*g* for 10 min at 4 °C. The IgG (ab150077, Abcam Inc., Cambridge, UK) and protein specific antibody anti-RUNX1 (ab92336, Abcam Inc., Cambridge, UK) were added into the two tubes, respectively, which were incubated at 4 °C overnight. The DNA–protein complex was subsequently precipitated by Protein Agarose/Sepharose, and centrifuged at 12,000×*g* for 5 min. The supernatant was discarded, and the nonspecific complex was washed to remove the cross-linking with incubation at 65 °C overnight. The DNA fragments were extracted and purified with phenol/chloroform, and the binding of RUNX1 and YAP promoter was then measured using RT-qPCR with YAP promoter region specific primers.

### Dual luciferase reporter gene assay

The wild type and mutant reporter plasmids of RUNX1-3′utr (pGL3-wt-RUNX1-3′utr, pGL3 -mut-RUNX1-3′utr) were designed and provided by Shanghai GenePharma Co. Ltd. (Shanghai, China). The Lewis lung cancer cells were co-transfected with antagomir NC and miR-21 antagomir with wt-RUNX1-3′utr and mut-RUNX1-3′utr respectively. After 48 h, the cells were collected and lysed. A dual luciferase reporter gene assay system (Promega Corporation, Madison, WI, USA) was employed for the detection of luciferase activity.

### Immunohistochemistry

The paraffin-embedded tumor tissues slices were dewaxed with xylene I and II (Shanghai Sangon Biotechnology Co. Ltd., Shanghai, China) for 10 min, rehydrated with 100%, 95% and 70% gradient ethanol (Shanghai Sangon Biotechnology Co. Ltd., Shanghai, China) for 2 min each. Next, the tissues were immersed in 3% H_2_O_2_ for 10 min and antigen retrieval was performed under high pressure in a pressure cooker for 90 s. The tissues were allowed to cool down to room temperature and then cut into slices, which were blocked with 5% bovine serum albumin (BSA), incubated at 37 °C for 30 min, added with 50 μL RUNX1 rabbit polyclonal antibody (ab92336, Abcam Inc., Cambridge, UK) and YAP rabbit monoclonal antibody (ab52771, Abcam Inc., Cambridge, UK) for incubation at 4 °C overnight. The following day, after rinsing with PBS for 2 min, the slices were added with 50 μL HRP labeled goat anti-rabbit antibody (ab6721, Abcam Inc., Cambridge, UK), incubated at 37 °C for 30 min and added with single antigen beads (SAB). Then, the slices were added with diaminobenzidine (DAB) solution (Fuzhou Maixin Biotechnology Development Co. Ltd. Fuzhou, China) for color development, re-stained with hematoxylin (Sigma-Aldrich, SF, CA, USA) for 5 min, and finally observed and photographed under an optical microscope (XSP-36, Bostar Optical Instruments Co., Ltd, Shenzhen, China). A total of 100 cells in each field were counted in 5 randomly selected high power visual fields, and the mean proportion of positive cells was calculated.

### RT-qPCR

Total RNA content in tissues or cells was extracted using the RNeasy Mini kits (Qiagen company, Hilden, Germany). The cDNA was synthesized using reverse transcription kits (RR047A, Takara Bio Inc., Otsu, Shiga, Japan) and the miRNA first strand cDNA synthesis first (tailing reaction) kits (B532451-0020, Shanghai Sangon Biotechnology Co. Ltd., Shanghai, China). RNA loading was performed using the SYBR^®^ Premix Ex Taq™ II (Perfect Real Time) kit (DRR081, Takara Bio Inc., Otsu, Shiga, Japan). RT-qPCR was carried out with a real-time PCR instrument (ABI 7500, ABI Company, Oyster Bay, NY). The general negative primer for miRNA and the upstream primer for glyceraldehyde-3-phosphate dehydrogenase (GAPDH) were provided by miRNA First Strand cDNA Synthesis (Tailing Reaction) kit, and the other primers were synthesized by Shanghai Sangon Biotechnology Co. Ltd. (Shanghai, China) (Table 1). The Ct value of each well was recorded. GAPDH or U6 were regarded as the internal references, and the relative expression of each gene was calculated using the 2^−ΔΔCt^ method.

**Table 1:** The primer sequences of RT-qPCR

Gene	Primer sequences
miR-21 (hsa)	F: GGACTAGCTTATCAGACTG
	R: CATCAGATG CGTTGCGTA
miR-21 (mus)	F: GACATCGCATGGCTGTACCA
	R: CCATGAGATTCAACAGTCAACATC
RUNX1	F: TGATGGCTGGCAATGATGAA
	R: TGCGGTGGGTTTGTGAAGAC
YAP	F: CGCTCTTCAACGCCGTCA
	R: AGTACTGGCCTGTCGGGAGT
ARG-1	F: CTCCAAGCCAAAGTCCTTAGAG
	R: AGGAGCTGTCATTAGGGACATC
iNOS	F: CCAAGCCCTCACCTACTTCC
	R: CTCTGAGGGCTGACACAAGG
GAPDH	F: TGAAGCAGGCATCTGAGGG
	R: CGAAGGTGGAAGAGTGGGAG
U6	F: CTCGCTTCGGCAGCACA
	R: AACGCTTCACGAATTTGCGT

RT-qPCR, reverse transcription quantitative polymerase chain reaction; RUNX1, runt-related transcription factor 1; YAP, yes-associated protein; ARG-1, Arginase-1; iNOS, inducible nitricoxide synthase; GAPDH, glyceraldehyde-3-phosphate dehydrogenase; F, forward ,R, reverse

### Western blot analysis

Total protein content in tissues or cells was extracted using radioimmunoprecipitation assay (RIPA) containing phenylmethylsulfonyl fluoride (PMSF). A bicinchoninic acid (BCA) kit was applied to measure the total protein concentration. A total of 50 μg protein was dissolved in 2×sodium dodecyl sulfate (SDS) sample buffer and boiled at 100 °C for 5 min. The protein was separated with sodium dodecyl sulfate–polyacrylamide electrophoresis (SDS-PAGE) and transferred onto a polyvinylidene fluoride (PVDF) membrane, and then sealed with 5% skim milk solution at room temperature for 1 h. The membrane was subsequently incubated overnight at 4 °C with the following primary antibodies purchased from Abcam (Cambridge, UK): rabbit anti-RUNX1 (ab92336), rabbit anti-YAP (ab52771), rabbit anti-ARG-1 (ab133543), iNOS (ab3523), and GAPDH (ab181602) as internal reference. Then, the membrane was washed three times with tris-buffered saline with Tween 20 (TBST) for 10 min each time. The membrane was then incubated with horseradish peroxidase (HRP) labeled goat anti-rabbit IgG H&L (ab6721, Abcam, Cambridge, UK) for 1 h, developed with an enhanced chemiluminescence (ECL) fluorescence test kit (BB-3501, Amersham, Chicago, Illinois, USA), and exposed in a gel imager. The membrane was digitally photographed using the Bio-Rad image analysis system (Bio-Rad Laboratories, Hercules, CA, USA), and analyzed with the Quantity One v4.6.2 software. The relative expression of the proteins was expressed by the ratio of gray value of each protein to that of internal reference GAPDH.

### Statistical analysis

Statistical analyses were conducted using the SPSS 21.0 statistical software (IBM, Armonk, NY, USA). Measurement data were summarized by mean ± standard deviation. When conforming to normal distribution and homogeneity, data between two unpaired groups were compared by unpaired t-test. Measurement data among multiple groups were compared by one-way analysis of variance (ANOVA) with Tukey’s post hoc test. Data comparison among multiple groups at different time points were conducted using repeated measurement ANOVA with Bonferroni’s post hoc test. Pearson’s correlation analysis was used to analyze the relationship between indicators. Measurement data were represented by examples, and verified using the Chi square test. A value of *p *< 0.05 indicated statistical significance.

## Results

### Down-regulation of miR-21 inhibits the immunosuppressive ability of MDSCs in lung cancer

**Fig. 1:**
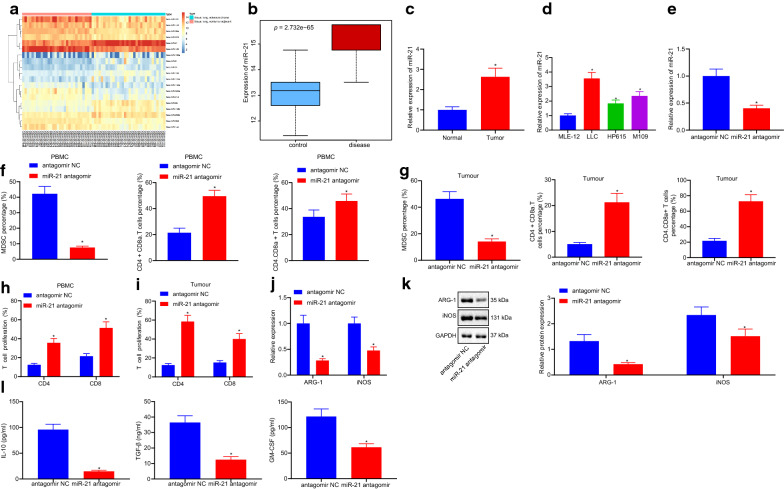
Downregulation of miR-21 inhibits the immunosuppressive ability of MDSCs in lung cancer. **a** heat map of 19 different miRNAs of dataset GSE63805; **b** box line map of miR-21 of dataset GSE63805, the blue box on the left shows the expression of normal samples, and the red box on the right shows the expression of lung cancer samples (p = 2.732e−65); **c**, **d**, miR-21 expression in lung cancer cells analyzed by RT-qPCR (n = 57), * *p *< 0.05 vs. the normal cells; # *p *< 0.05 vs. the MLE-12; **e** the relative expression of miR-21 measured by RT-qPCR; **f**, **g**, the proportion of MDSCs, Th, CTL in the peripheral blood (**f**) and tumor tissues (**g**) of mice measured by a flow cytometer; **h** CD4^+^ or CD8^+^ T cells labeled with CFSC detected by flow cytometry; **i**, **j**, the inhibition effect of MDSCs on Th, CTL detected by T cell proliferation analysis; **k** the expression of ARG-1 and iNOS measured by RT-qPCR; **l** the expression of ARG-1 and iNOS protein measured by western blot analysis; **m** the expression of IL-10, TGF-β and GM-CSF analyzed by ELISA. * *p *< 0.05 vs. antagomir NC. The data in the figures were all measurements, expressed as mean ± standard deviation; n = 12. Data between two unpaired groups were compared by unpaired t-test. One-way ANOVA with Tukey’s post hoc test was used to compare data among multiple groups. The experiment was repeated three times

Differential analyses of the GSE63805 dataset using the R language revealed 19 differentially expressed miRNAs, among which miR-21 exhibited the most significant expression (Fig. 1a). Thus, the role and mechanism of miR-21 on the immunosuppressive ability of MDSCs was explored in lung cancer.

Firstly, in order to determine the expression patterns of miR-21 in lung cancer, a box line map was plotted by extracting the expression data of miR-21 of dataset GSE63805, which revealed that miR-21 was over-expressed in the lung cancer samples (Fig. 1b). RT-qPCR analysis showed that, compared with normal tissues, the expression levels of miR-21 in lung cancer tissues were higher. Compared with the mouse lung epithelial cells-12 (MLE-12), miR-21 expression was also found to be up-regulated in all of the lung cancer cell lines (*p *< 0.05) (Fig. 1c, d). Statistical analysis of the relationship between miR-21 expression and clinical indicators of lung cancer patients demonstrated that the expression level of miR-21 was closely related to tumor size, tumor node metastasis (TNM) stage, smoking history, and presence of lymph node metastasis (Table 2).

**Table 2:** The relationship between miR-21 expression and cliniccopathological features of lung cancer patients

Clinicopathological features	Cases (n=30)	miR-21 expression		P value
		High	Low	
Age (years)				0.7047
> 60	23	12	11	
≤ 60	34	16	18	
Gender				0.1885
Male	29	17	12	
Female	28	11	17	
Tumor size (cm)				0.0030
≥ 3.0	33	22	11	
< 3.0	24	6	18	
TNM stage				0.0277
I–II	21	6	15	
IIIa	36	22	14	
Smoking history				0.0167
Smokers	31	20	11	
Non-mokers	26	8	18	
Lymph node metastasis				0.0074
Positive	24	17	7	
Negative	33	11	22	
Histological tumor type				0.2828
Squamous cell carcinoma	19	11	8	
Adenocarcinoma	21	12	9	
Small cell lung cancer	13	4	9	
Large cell lung cancer	4	1	3	

Meanwhile, expression levels of miR-21 in mice treated with miR-21 antagomir were found to be lower than those in mice treated with antagomir NC (*p *< 0.05) (Fig. 1e). In addition, the proportion of MDSCs with CD11b^+^Gr-1^+^ in the peripheral blood was elevated in the modeled mice. Next, peripheral blood and tumor tissues were further evaluated to uncovered the effect of miR-21 on the immunosuppression of lung cancer. Flow cytometry was applied to analyze the proportion of MDSCs, T helper (Th) and cytotoxic T lymphocyte (CTL) in the peripheral blood and tumor tissues of mice treated with miR-21 antagomir. The results indicated that, compared with antagomir NC, the proportion of MDSCs was notably decreased, while the proportion of Th and CTL were increased in mice treated with miR-21 antagomir (*p *< 0.05) (Fig. 1f, g). In addition, CD4^+^ or CD8^+^ T cells labeled with CFSC were also detected using flow cytometry (Fig. 1h), which revealed that, compared with mice treated with antagomir NC, MDSCs in the peripheral blood and tumor tissues of mice injected with miR-21 antagomir markedly reduced the inhibition of Th and CTL proliferation (*p *< 0.05) (Fig. 1i, j). Moreover, the expression patterns of MDSCs functional markers ARG-1, iNOS were measured by RT-qPCR and Western blot analysis, which showed that the mice injected with miR-21 antagomir exhibited significantly reduced expressions of ARG-1, iNOS compared with the NC (*p *< 0.05) (Fig. 1k, l). ELISA analysis showed that IL-10, TGF-β and GM-CSF levels in the mice injected with miR-21 antagomir were much lower than the NC (Fig. 1m). Overall, these findings indicated that down-regulation of miR-21 can retard the immunosuppressive ability of MDSCs on lung cancer.

### miR-21 promotes the expression of YAP by inhibiting RUNX1

**Fig. 2:**
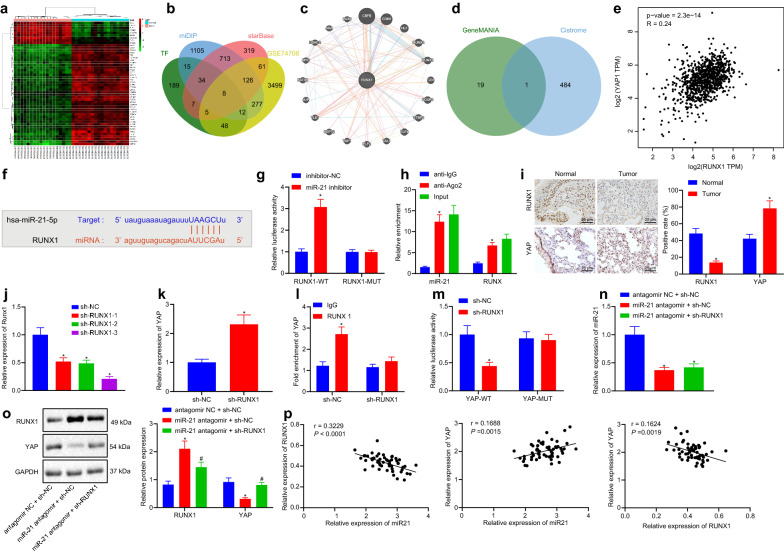
miR-21 upregulates YAP expression via inhibiting RUNX1. **a** heat map of 50 genes of dataset GSE74706; **b** downstream genes of miR-21 and significant differentially expressed genes of dataset GSE74706 predicted by mirDIP and starBase and the Venn map of human transcription factor obtained from Cistrome, with BCL11A, E2F3, ERG, ETS1, KLF5, MEF2A, RUNX1 and TTF2 as the intersection genes; **c** PPI map of RUNX1 and its related genes obtained from GeneMANIA; **d** Venn map of genes related to RUNX1 obtained from GeneMANIA and target genes of RUNX1 predicted by Cistrome in lung adenocarcinoma, with YAP1 (YAP) as the only intersection gene; **e** correlation of RUNX1 and YAP1 in lung adenocarcinoma and squamous cell carcinoma of TCGA database analyzed by GEPIA (P = 2.3e−14); **f** binding site sequences of miR-21 and RUNX1; **g** relationship between miR-21 and RUNX1 measured by dual luciferase reporter gene assay, * *p *< 0.05 vs. miR-21; **h** binding of miR-21 and RUNX1 analyzed by RIP, * *p *< 0.05 vs. anti-IgG1; **i** the expression of RUNX1 and YAP in tumor tissues measured by immunohistochemistry, * *p *< 0.05 vs. normal mice; **j** the expression of RUNX1 after transfection in tissues; **k** the expression of YAP when RUNX1 was silenced; **l** the binding of RUNX1 and YAP detected by ChIP assay, * *p *< 0.05 vs. IgG; **m** the binding of RUNX1 and YAP verified by dual luciferase reporter gene assay; **n** the expression of miR-21 in cells measured by RT-qPCR; **o** the expression of RUNX1 and YAP in cells measured by western blot analysis; **p** the correlation of miR-21 and RUNX1, miR-21 and YAP, RUNX1 and YAP analyzed by Pearson. Measurement data were expressed as mean ± standard deviation. When data were homogeneous and of normal distribution, data between two unpaired groups were compared by unpaired t-test. Measurement data among multiple groups were checked by ANOVA with Tukey’s post hoc test. Pearson’s correlation analysis was used to analyze the relationships between miR-21, RUNX1 and YAP

To further examine the downstream regulatory mechanism of miR-21, 2290 downstream target genes of miR-21 were retrieved through mirDIP, 1273 downstream target genes through starBase, and 4036 differentially expressed genes in lung cancer following analyses of the GSE74706 dataset in the GEO database (Fig. 2a). The predicted downstream target gene of miR-21 was compared with the differentially expressed gene and the human transcription factors in Cistrome, which yielded a total of 8 transcription factors with significant differences in the downstream target genes of miR-21 in lung cancer (Fig. 2b). In addition, GeneMANIA revealed 20 genes related to RUNX1 (Fig. 2b), while Cistrome predicted 485 highly-correlated target genes of RUNX1 in lung adenocarcinoma. The intersection of GeneMANIA and Cistrome results was observed to identify the key downstream target of RUNX1, which proved to be the YAP1 gene (Fig. 2d). Through further GEPIA analysis of lung adenocarcinoma and lung squamous cell carcinoma data in the TCGA database, RUNX1 and YAP1 were found to be negatively-correlated (Fig. 2e). Meanwhile, the starbase website predicted that miR-21 could inhibit the expression of transcription factor RUNX1 (Fig. 2f). Therefore, we speculated that miR-21 might regulate the YAP expression by inhibiting the RUNX1 factor in mice.

Additional microarray-based analyses suggested the presence of binding sites between miR-21 and RUNX1 (Fig. 2f). At the same time, the targeting relationship between miR-21 and RUNX1 was confirmed using a dual luciferase reporter gene assay, which demonstrated that the fluorescence intensity of cells co-transfected with miR-21 inhibitor and RUNX1-WT was higher than that in cells treated with miR-21 NC only (*p *< 0.05), while there were no such differences in the cells co-transfected with miR-21 inhibitor and RUNX1-WT (P > 0.05) (Fig. 2g). The combination of miR-21 and RUNX1 detected by RIP assay indicated greater enrichment of miR-21 and RUNX1 in cells treated with Ago2 group relative to the cells treated with IgG (*p *< 0.05) (Fig. 2h).

Furthermore, immunohistochemistry results showed that, compared with the NC, the expression levels of RUNX1 in tumor bearing mice was lower, while those of YAP was increased (*p *< 0.05) (Fig. 2i). Then, RUNX1 was knocked-down to specifically interfere with the small RNAs, and the interference efficiency was detected. Results showed that, compared with the cells treated with sh-NC, the expression levels of RUNX1 were notably decreased in cells treated with sh-RUNX1-1, sh-RUNX1-2, and sh-RUNX1-3 (*p *< 0.05), indicating that the low expression vector was successfully-transfected. The expression of RUNX1 in cells treated with sh-RUNX1-3 was found to be decreased most significantly, and was thus chosen for subsequent experimentation (Fig. 2j).

The results of RT-qPCR further showed that the mRNA expression levels of YAP in the cells transfected with sh-RUNX1 were higher than in the NC cells (*p *< 0.05) (Fig. 2k). In addition, ChIP assay indicated that, compared with the cells treated with IgG, the DNA of YAP gene promoter in cells transfected with RUNX1 was remarkably increased (*p *< 0.05), indicating that the transcription factor RUNX1 regulated the expression of YAP in the gene promoter region. Besides, when RUNX1 was knocked-down in Lewis lung cancer cells, the enrichment of RUNX1 on the YAP gene promoter was found to be reduced (*p *< 0.05) (Fig. 2l). According to the dual luciferase reporter gene assay, the fluorescence intensity of cells co-treated with sh-RUNX1 and YAP-WT was much higher than in the cells treated with sh-NC (*p *< 0.05), while there were no significant differences in the cells co-treated with sh-RUNX1 and YAP-MUT (*p* > 0.05) (Fig. 2m). Moreover, the expression patterns of miR-21 were detected using RT-qPCR (Fig. 2n), while those of RUNX1 and YAP (Fig. 2o) were assessed by Western blot analysis respectively. These assays showed that the down-regulation of YAP mediated by miR-21 antagomir could be reversed by sh-RUNX1 (*p *< 0.05). Finally, Pearson correlation analysis of miR-21 and RUNX1, miR-21 and YAP, RUNX1 and YAP showed that miR-21 expression was negatively-correlated with that of RUNX1, RUNX1 and YAP, whereas the expressions of miR-21 and YAP were positively-correlated. Consequently, these findings indicated that miR-21 up-regulated YAP through inhibition of RUNX1.

### miR-21 regulates the immunosuppressive ability of MDSCs against lung cancer via promoting the expression of YAP mediated by RUNX1

**Fig. 3:**
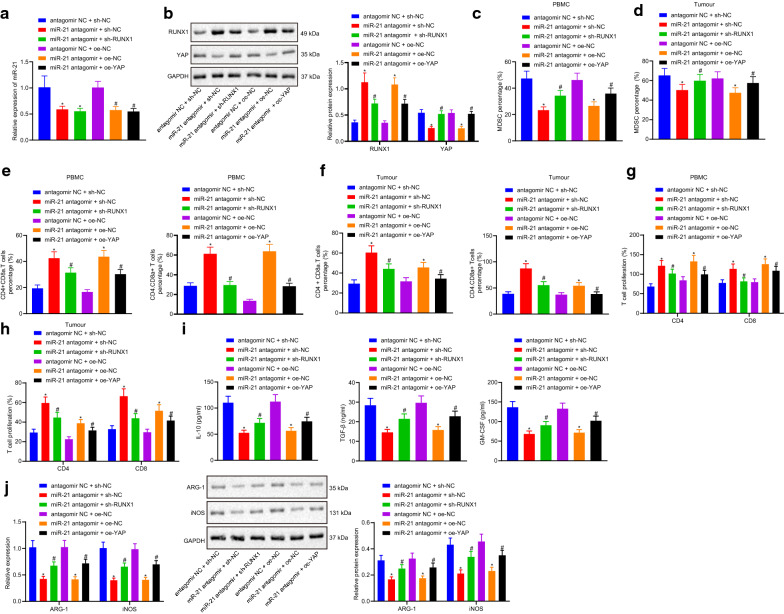
The miR-21/RUNX1/YAP axis regulated the immunosuppressive ability of MDSCs in lung cancer. **a** the expression of miR-21 analyzed by RT-qPCR; **b** the expression of RUNX1 and YAP measured by western blot analysis; **c**, **d**, the proportion of MDSCs in peripheral blood and tumor tissues of mice detected by flow cytometry; **e**, **f** the proportion of Th and CTL in peripheral blood and tumor tissues measured by flow cytometry; **g**, **h** the inhibitory effect of MDSCs on Th and CTL in peripheral blood and tumor tissues of mice analyzed by T cell proliferation assay; **i** the expression of IL-10, TGF-β and GM-CSF were detected by ELISA; **j** the expression of ARG-1 and iNOS measured by RT-qPCR and western blot analysis. Measurement data were expressed as mean ± standard deviation. * *p *< 0.05 vs. cells co-treated with antagomir NC and sh-NC or antagomir NC and oe-NC, # *p *< 0.05 vs. cells co-treated with miR-21 antagomir and sh-NC or miR-21 antagomir and oe-NC. Measurement data among multiple groups were compared by one-way ANOVA with Tukey’s post hoc test. The experiment was repeated three times

RT-qPCR showed that the expression levels of miR-21 in the cells co-treated with miR-21 antagomir and sh-NC, miR-21 antagomir and sh-RUNX1 were lower than those in cells treated with antagomir NC and sh-NC (*p *< 0.05). Furthermore, the cells co-treated with miR-21 antagomir and oe-NC, or miR-21 antagomir and oe-YAP exhibited lower miR-21 expression levels relative to cells treated with antagomir NC and oe-NC (Fig. 3a). Western blot analysis showed that the levels of RUNX1 were notably increased, while those of YAP were lower in the cells co-transfected with miR-21 antagomir and sh-NC compared to cells co-treated with antagomir NC and sh-NC. Compared with the cells co-treated with miR-21 antagomir and sh-NC, the levels of RUNX1 were decreased, while those of YAP were dramatically increased in the cells co-transfected with miR-21 antagomir and sh-RUNX1. Compared with the cells co-treated with antagomir NC and oe-NC, the levels of RUNX1 were increased, whereas YAP levels were notably decreased in the cells co-transfected with miR-21 antagomir and oe-NC. The expression levels of RUNX1 were much lower, while the expression of YAP was much higher in the cells co-transfected with miR-21 antagomir and oe-YAP relative to those in the cells co-treated with miR-21 antagomir and oe-NC (*p *< 0.05) (Fig. 3b).

In addition, flow cytometry was performed to measure the proportion of MDSCs, Th and CTL in the peripheral blood and tumor tissues of the mice, which revealed that the proportion of MDSCs was decreased, while the proportion of Th and CTL were dramatically increased in the cells co-transfected with miR-21 antagomir and sh-NC compared to the cells co-treated with antagomir NC and sh-NC, whereas the opposite trends were observed in cells co-transfected with miR-21 antagomir and sh-RUNX1 in relation to cells co-treated with miR-21 antagomir and sh-NC. The proportion of MDSCs was also found to be remarkably reduced, while the proportion of Th and CTL were higher in the cells co-transfected with miR-21 antagomir and oe-NC than that in the cells co-treated with antagomir NC and oe-NC. In contrast to the cells co-treated with miR-21 antagomir and oe-NC, the proportion of MDSCs was highly increased, whereas the proportion of Th and CTL were decreased in the cells co-transfected with miR-21 antagomir and oe-YAP (*p *< 0.05) (Fig. 3c–f).

Furthermore, the MDSCs in the peripheral blood and tumor tissues in the mice treated with both miR-21 antagomir and sh-NC inhibited Th and CTL proliferation compared with that in the mice treated with both antagomir NC and sh-NC, which was relieved by the addition of sh-RUNX1. Compared with the mice co-treated with antagomir NC and oe-NC, the MDSCs in peripheral blood and tumor tissues of mice co-treated with miR-21 antagomir and oe-NC inhibited the proliferation of Th and CTL, while the opposite was true in MDSCs in peripheral blood and tumor tissues of mice injected with miR-21 antagomir and oe-YAP compared with mice treated with both miR-21 antagomir and oe-NC (*p *< 0.05) (Fig. 3g–h).

ELISA assay showed that the expression levels of IL-10, TGF-β and GM-CSF in cells co-treated with miR-21 antagomir and sh-NC were dramatically lower than those in cells co-transfected with antagomir NC and sh-NC, while the opposite trends were observed in cells treated with miR-21 antagomir and sh-RUNX1 in comparison to miR-21 antagomir and sh-NC. The levels of IL-10, TGF-β and GM-CSF were also decreased in the cells treated with miR-21 antagomir and oe-NC relative to the cells treated with both antagomir NC and oe-NC, whereas the opposite results were witnessed in cells treated with both miR-21 antagomir and oe-YAP than that co-treated with miR-21 antagomir and oe-NC (*p *< 0.05) (Fig. 3i).

Then, the mRNA and protein expression patterns of ARG-1 and iNOS, the two functional markers of MDSCs, were analyzed using RT-qPCR and Western blot analysis. The results showed lower mRNA and protein expression levels of ARG-1 and iNOS in the cells transfected with both miR-21 antagomir and sh-NC compared to cells co-treated with antagomir NC and sh-NC. However, these expression levels were found to be elevated in the cells transfected with both miR-21 antagomir and sh-RUNX1 compared with the cells co-treated with miR-21 antagomir and sh-NC. Meanwhile, decreased expression levels were observed in the cells co-transfected with miR-21 antagomir and oe-NC when compared with the cells treated with both antagomir NC and oe-NC, which was reversed following co-treatment with miR-21 antagomir and oe-YAP (Fig. 3j). Consequently, these findings suggested that miR-21 can regulate YAP by regulating the expression of RUNX1 to mediate the immunosuppressive ability of MDSCs in lung cancer.

### Immunosuppressive effect of the miR-21/RUNX1/YAP axis in regulating the cell cycle and apoptosis of MDSCs in vitro

**Fig. 4:**
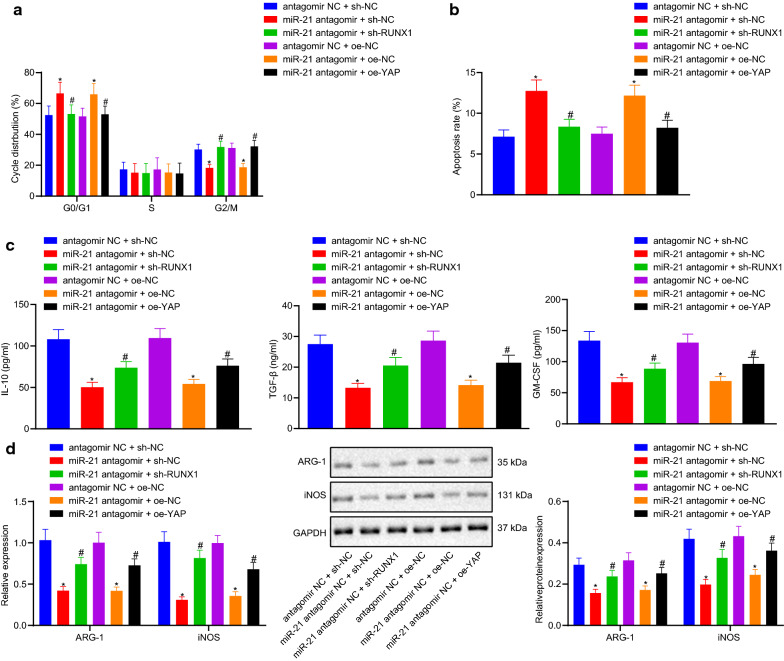
The effect of miR-21/RUNX1/YAP axis on immunosuppressive ability of MDSCs in lung cancer in vitro. **a** the proportion of MDSCs in peripheral blood cells before and after magnetic bead sorting; **b** the cell cycle of MDSCs analyzed by a flow cytometer; **c** apoptosis map of MDSCs after 24 h treatment; **d** the expression of IL-6 and other effectors in MDSCs measured by ELISA; **e** the expression of ARG-1 and iNOS detected by RT-qPCR and western blot analysis. Measurement data were expressed as mean ± standard deviation. * *p *< 0.05 vs. cells co-treated with antagomir NC and sh-NC or antagomir NC and oe-NC, # *p *< 0.05 vs. cells co-treated with miR-21 antagomir and sh-NC or miR-21 antagomir and oe-NC. Measurement data among multiple groups were compared by ANOVA with Tukey’s post hoc test. The experiment was repeated three times

Furthermore, the effects of miR-21/RUNX1/YAP axis on MDSCs cycle, apoptosis and key immunosuppressive molecules were studied in vitro. MDSCs purity of the CD11b^+^Gr-1^+^ phenotype was found to be raised from 21.2% to 96.6% after magnetic bead separation, indicating that the magnetic bead separation successfully enriched the MDSCs with CD11b^+^Gr-1^+^ phenotype, which were then used for the following in vitro experimentation (Fig. 4a).

Flow cytometry was then applied to analyze the ability of the miR-21/RUNX1/YAP axis to regulate the cell cycle and apoptosis of MDSCs. The distribution of MDSCs in different states was assessed using Annexin V and PI double staining. The staining results illustrated that the apoptosis rate of MDSCs co-treated with miR-21 antagomir and sh-NC, miR-21 antagomir and oe-NC was enhanced, accompanied by more cells at the G0/G1 phase, fewer cells at the G2/M phase, with no differences in cells at the S phase in comparison to those in MDSCs co-transfected with antagomir NC and sh-NC, antagomir NC and oe-NC, respectively. On the contrary, compared with MDSCs co-treated with miR-21 antagomir and sh-NC, miR-21 antagomir and oe-NC, the apoptosis rate of MDSCs co-transfected with miR-21 antagomir and sh-RUNX1, miR-21 antagomir and oe-YAP was notably decreased, and there were fewer cells at the G0/G1 phase, more cells at the G2/M phase, with no differences in cells at the S phase correspondingly (*p *< 0.05) (Fig. 4b, c).

Additionally, the results of ELISA indicated that, compared with the cells co-treated with antagomir NC and sh-NC, the expression levels of IL-10, TGF-β and GM-CSF in the cells co-treated with miR-21 antagomir and sh-NC were decreased, compared with the latter, while these expression levels were increased in the cells co-treated with miR-21 antagomir and sh-RUNX1 (*p *< 0.05). The levels of IL-10, TGF-β and GM-CSF in the cells co-treated with miR-21 antagomir and oe-NC were much lower than in the cells transfected with antagomir NC and oe-NC in combination, while the opposite trends were observed in the cells co-treated with miR-21 antagomir and oe-YAP compared to those in the cells co-treated with miR-21 antagomir and oe-NC (*p *< 0.05) (Fig. 4d).

RT-qPCR and Western blot analysis revealed that the mRNA and protein expression levels of ARG-1 and iNOS in the cells treated with both miR-21 antagomir and sh-NC were lower than those in the cells treated with both antagomir NC and sh-NC, whereas these expression levels were found to be notable higher in the cells co-treated with miR-21 antagomir and sh-RUNX1 compared to the former (*p *< 0.05). In comparison with cells transfected with antagomir NC and oe-NC, the cells treated with miR-21 antagomir and oe-NC exhibited lower mRNA and protein expression levels of ARG-1 and iNOS. Compared with the cells transfected with miR-21 antagomir and oe-NC in combination, mRNA and protein expression levels of ARG-1 and iNOS in the cells co-treated with antagomir NC and oe-NC, miR-21 antagomir and oe-YAP were higher (Fig. 4e). Collectively, these findings suggested that miR-21/RUNX1/YAP axis promote the cycle and apoptosis of MDSCs and inhibit the key immunosuppressive molecules.

### miR-21 promotes tumor development through promoting the expression of YAP mediated by RUNX1 in vivo

**Fig. 5:**
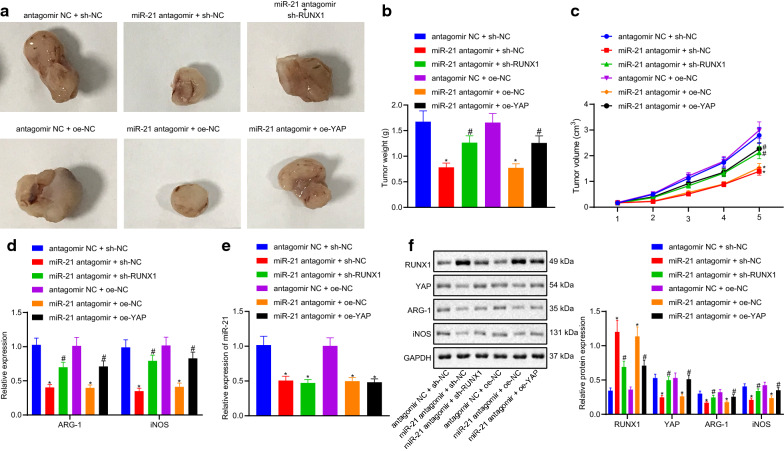
The effect of miR-21/RUNX1/YAP axis on the immunosuppressive ability of lung cancer xenografted tumor in vivo. **a** representative image of xenotransplantation tumor formation in nude mice; **b** tumor volume of mice; **c** tumor mass of mice; **d** the expression of ARG-1 and iNOS analyzed by RT-qPCR; **e** the expression of miR-21 detected by RT-qPCR; **f** the expression of RUNX1, YAP, ARG-1 and iNOS measured by western blot analysis. Measurement data were expressed as mean ± standard deviation. * *p *< 0.05 vs. cells co-treated with antagomir NC and sh-NC or antagomir NC and oe-NC, # *p *< 0.05 vs. cells co-treated with miR-21 antagomir and sh-NC or miR-21 antagomir and oe-NC. Measurement data among multiple groups were checked by ANOVA with Tukey’s post hoc test. Data comparison among multiple groups at different time points was conducted using repeated measurement ANOVA with Bonferroni’s post hoc test. The experiment was repeated three times

The average tumor volume and weight in mice co-treated with miR-21 antagomir and sh-NC were found to be lower than that in mice injected with antagomir NC and sh-NC. However, compared with the mice inoculated with both miR-21 antagomir and sh-NC, the average tumor volume and weight in mice inoculated with both miR-21 antagomir and sh-RUNX1 were both increased (*p *< 0.05). Compared with the mice co-treated with antagomir NC and oe-NC, the average tumor volume and weight in mice co-treated with miR-21 antagomir and oe-NC were both decreased, compared with the latter, the average tumor volume and weight in mice co-treated with miR-21 antagomir and oe-YAP were both increased (*p *< 0.05) (Fig. 5a–c).

The results of RT-qPCR showed that the expression levels of ARG-1 and iNOS in the mice treated with both miR-21 antagomir and sh-NC were lower than that in the mice treated with both antagomir NC and sh-NC, but those in the mice co-treated with miR-21 antagomir and sh-RUNX1 were notably higher than the former (*p *< 0.05). The levels of ARG-1 and iNOS in the mice co-treated with miR-21 antagomir and oe-NC were much lower than in the mice injected with antagomir NC and oe-NC in combination. However, but the expression levels of ARG-1 and iNOS in the mice co-treated with miR-21 antagomir and oe-YAP were much higher than in the mice co-treated with miR-21 antagomir and oe-NC (*p *< 0.05) (Fig. 5d).

RT-qPCR results showed that the expression levels of miR-21 in the mice co-treated with miR-21 antagomir and sh-NC, miR-21 antagomir and sh-RUNX1 were lower than those in the mice co-treated with antagomir NC and sh-NC (*p *< 0.05). Compared with the mice co-treated with antagomir NC and oe-NC, the levels of miR-21 in the mice inoculated with both miR-21 antagomir and oe-NC, miR-21 antagomir and oe-YAP were unaffected (*p *< 0.05) (Fig. 5e).

Western blot analysis indicated that the protein levels of RUNX1 were notably increased, while the protein levels of YAP, ARG-1 and iNOS were all remarkably decreased in the mice co-treated with miR-21 antagomir and sh-NC compared to those in the mice co-treated with antagomir NC and sh-NC, while the opposite was true for the mice co-treated with miR-21 antagomir and sh-RUNX1 compared with miR-21 antagomir and sh-NC (*p *< 0.05). Compared with the mice co-treated with antagomir NC and oe-NC, the protein levels of RUNX1 were increased, whereas those of YAP, ARG-1 and iNOS were notably decreased in the mice co-transfected with miR-21 antagomir and oe-NC, but the results were opposite in the mice co-treated with miR-21 antagomir and oe-YAP than the latter (*p *< 0.05) (Fig. 5f). In conclusion, these findings indicated that miR-21 promotes tumor development by elevating the expression of RUNX1-mediated YAP.

## Discussion

As the leading cause of cancer-related deaths among men and women, the occurrence of lung cancer is closely related to smoking and the use of tobacco products, in addition to environmental factors such as air pollution [21]. Advancements in lung cancer treatment incorporating the use of chemotherapy and targeted therapies have improved the outcomes of the disease, however, the overall prognoses with respect to long term survival remains unsatisfactory [22]. At the same time, our understanding of the molecular mechanisms of tumor immunology, especially the inhibition of anti-tumor immune response mediated by immune synapses or immune checkpoints, has grown rapidly in the past decade owing to the hard-done work of our peers [23]. Aiming to expand on this, the current study set out to explore the specific molecular mechanism of miR-21 on the immunosuppressive ability of MDSCs against lung cancer.

A number of miRNAs possess the ability to regulate the differentiation, maturation and function of immune cells, and as a result serve as important contributors in maintaining cellular homeostasis and the development of distinct physiological systems [24]. In the current study, our findings revealed the existence of one such miRNA, namely miR-21, which exhibited up-regulated expression levels in lung cancer tissues. Augmented levels of miR-21 have been documented in lung cancer by Wu et al, which are very much in accordance with our results [25]. Similarly, miR-21 was reported to be highly-expressed in NSCLC, and to further regulate invasion and chemo-sensitivity of the cancer by mediating the SMAD7 gene [26]. In addition, down-regulation of miR-21 has been previously shown to inhibit the proliferation and migration of non-small cell lung cancer cells via mediation of programmed cell death [27]. However, it is not clear whether and how miR-21 participates in the differentiation and functional regulation of MDSCs. Furthermore, extensive experimentation in our study demonstrated that down-regulation of miR-21 can inhibit the immunosuppressive ability of MDSCs to lung cancer. Moreover, other studies have shown that miR-21 can also regulate the immune resistance of myelogenous suppressor cells to tumor [28].

Additionally, our findings revealed that the RUNX1 transcription factor was the downstream target of miR-21 with the help of mirDIP and starBase databases. Indeed, RUNX1 is an important regulator of hematopoiesis, and also related with heightening of metastasis [29]. Meanwhile, another study uncovered that miR-9 regulated MDSCs differentiation by targeting the RUNX1 factor, highlighting its transcription-based role in regulating MDSC differentiation and function [24]. Our results verified that RUNX1 was poorly-expressed in lung cancer tissues. Similarly, RUNX1 is down-regulated and negatively-correlated with MDSC-mediated immunosuppression in lung cancer [30]. Also, the Rasip1 gene is known to be regulated by RUNX1 to promote the migration of NSCLC [29]. Meanwhile, intersection results from GeneMANIA and Cistrome highlighted YAP as the downstream target of RUNX1 in lung cancer. Previous studies have also shown that RUNX1 can inhibit YAP expression to accelerate the occurrence of tumors and inhibit the expression of its target gene [17]. Highly-expressed levels of the YAP tumor protein have been previously found in NSCLC, suggesting an important role in regulating the growth and invasion of tumor cells [31]. Furthermore, YAP has been shown to promote tumor development by controlling the infiltration of MDSCs [32, 33]. As a result, our findings suggested that miR-21 can up-regulate the expression levels of YAP by targeting RUNX1 to regulate the immunosuppressive ability of MDSCs in lung cancer.

On the other hand, previous studies have indicated that over-expression of miR-21 leads to amplified invasion of MCF-7 cancer cells, and enhanced EGF-mediated invasion and TGF-β-mediated invasion in breast cancer [34]. In addition, miR-21 functions as an upstream regulator of IL-10 by targeting the 3′ untranslated region of IL-10 mRNA [35]. Recent studies have also demonstrated that miR-21 targets IL-10 mRNA, and further plays a proinflammatory role by retarding IL-10-expressing regulatory B cell (B10) differentiation [36]. Similarly, the ELISA results in our study indicated that silencing miR-21 diminished the expression levels of IL-10, TGF-β and GM-CSF, which was reserved by silencing RUNX1. All in all, our findings indicate that miR-21/RUNX1/YAP axis can promote the cycle and apoptosis of MDSCs, and thereby, inhibit the effect of key immunosuppressive molecules.

## Conclusions

**Fig. 6:**
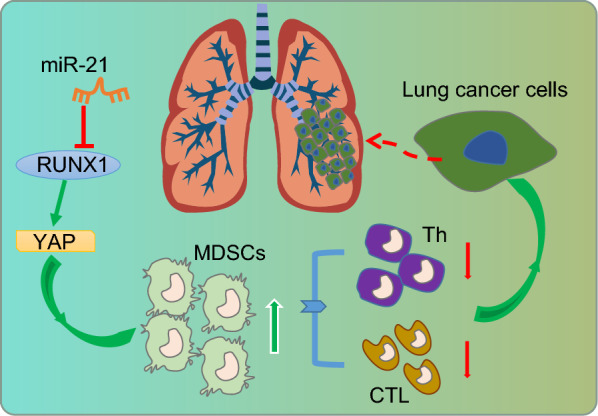
Schematic representation of the potential molecular mechanisms involved in miR-21 in immunosuppressive ability of MDSCs in lung cancer. In lung cancer, upregulated miR-21 inhibits the expression of downstream target gene RUNX1. Since the expression of YAP was mediated through binding with RUNX1 in the promoter region, so the downregulation of RUNX1 can upregulate the expression of YAP, thus inhibiting the immune resistance of MDSCs and promoting the development of tumors

Collectively, our findings demonstrated that up-regulation of miR-21 inhibits the expression of the downstream target RUNX1 in lung cancer. RUNX1 subsequently binds to the promoter region of the YAP gene to downregulate its expression. We conclude that miR-21 up-regulated the YAP expression by inhibiting the transcription factor RUNX1 to regulate the immunosuppressive ability of MDSCs against lung cancer (Fig. 6). Our findings not only improve the understanding of how miR-21 modulates the immunosuppressive ability of MDSCs in lung cancer, but also offer a potential prognostic marker and a therapeutic target in the form of miR-21 silencing for lung cancer.

## Data Availability

The authors confirm that the data supporting the findings of this study are available within the article

## Abbreviations

miR-21: microRNA-21
MDSCs: Myeloid-derived suppressor cells
Th: T helper
GEO: Gene Expression Omnibus
TCGA: The Cancer Genome Atlas
GEPIA: Gene Expression Profiling Interactive Analysis
DMEM: Dulbecco’s Modified Eagle Medium
PBS: Phosphate buffer saline
NC: Negative control
CFSE: Carboxyfluorescein diacetate, succinimidylester
PI: Propidium iodide
RIPA: Radio-immunoprecipitation assay
GAPDH: Glyceraldehyde-3-phosphate dehydrogenase
PMSF: Phenylmethylsulfonyl fluoride
BCA: Bicinchoninic acid
SDS: Sodium dodecyl sulfate
SDS-PAGE: Sodium dodecyl sulfate–polyacrylamide electrophoresis
PVDF: Polyvinylidene fluoride

## References

[1] Bray F, Ferlay J, Soerjomataram I, Siegel RL, Torre LA, Jemal A (2018). Global cancer statistics 2018: GLOBOCAN estimates of incidence and mortality worldwide for 36 cancers in 185 countries. CA Cancer J Clin.

[2] Cheung CHY, Juan HF (2017). Quantitative proteomics in lung cancer. J Biomed Sci.

[3] Yousefi M, Bahrami T, Salmaninejad A, Nosrati R, Ghaffari P, Ghaffari SH (2017). Lung cancer-associated brain metastasis: molecular mechanisms and therapeutic options. Cell Oncol (Dordr)..

[4] Asmar R, Rizvi NA (2015). Immunotherapy for advanced lung cancer. Cancer J.

[5] Kolahian S, Oz HH, Zhou B, Griessinger CM, Rieber N, Hartl D (2016). The emerging role of myeloid-derived suppressor cells in lung diseases. Eur Respir J.

[6] Bosiljcic M, Cederberg RA, Hamilton MJ, LePard NE, Harbourne BT, Collier JL (2019). Targeting myeloid-derived suppressor cells in combination with primary mammary tumor resection reduces metastatic growth in the lungs. Breast Cancer Res.

[7] Lan J, Li R, Yin LM, Deng L, Gui J, Chen BQ (2018). Targeting myeloid-derived suppressor cells and programmed death ligand 1 confers therapeutic advantage of ablative hypofractionated radiation therapy compared with conventional fractionated radiation therapy. Int J Radiat Oncol Biol Phys.

[8] Liu Y, Lai L, Chen Q, Song Y, Xu S, Ma F (2012). MicroRNA-494 is required for the accumulation and functions of tumor-expanded myeloid-derived suppressor cells via targeting of PTEN. J Immunol..

[9] Wang J, Yu F, Jia X, Iwanowycz S, Wang Y, Huang S (2015). MicroRNA-155 deficiency enhances the recruitment and functions of myeloid-derived suppressor cells in tumor microenvironment and promotes solid tumor growth. Int J Cancer.

[10] Tang TT, Liu BC (2019). Extracellular vesicles: opportunities and challenges for the treatment of renal fibrosis. Adv Exp Med Biol.

[11] Leonetti A, Assaraf YG, Veltsista PD, El Hassouni B, Tiseo M, Giovannetti E (2019). MicroRNAs as a drug resistance mechanism to targeted therapies in EGFR-mutated NSCLC: current implications and future directions. Drug Resist Updat..

[12] Ren W, Hou J, Yang C, Wang H, Wu S, Wu Y (2019). Extracellular vesicles secreted by hypoxia pre-challenged mesenchymal stem cells promote non-small cell lung cancer cell growth and mobility as well as macrophage M2 polarization via miR-21-5p delivery. J Exp Clin Cancer Res..

[13] Zhou B, Wang D, Sun G, Mei F, Cui Y, Xu H (2018). Effect of miR-21 on apoptosis in lung cancer cell through inhibiting the PI3K/Akt/NF-kappaB signaling pathway in vitro and in vivo. Cell Physiol Biochem.

[14] de Bruijn M, Dzierzak E (2017). Runx transcription factors in the development and function of the definitive hematopoietic system. Blood.

[15] Maeda R, Sato S, Obata S, Ohno T, Hashiya K, Bando T (2019). Molecular characteristics of DNA-Alkylating PI polyamides targeting RUNX transcription factors. J Am Chem Soc.

[16] Sood R, Kamikubo Y, Liu P (2017). Role of RUNX1 in hematological malignancies. Blood.

[17] Kulkarni M, Tan TZ, Syed Sulaiman NB, Lamar JM, Bansal P, Cui J (2018). RUNX1 and RUNX3 protect against YAP-mediated EMT, stem-ness and shorter survival outcomes in breast cancer. Oncotarget..

[18] Kim MH, Kim CG, Kim SK, Shin SJ, Choe EA, Park SH (2018). YAP-Induced PD-L1 expression drives immune evasion in BRAFi-resistant melanoma. Cancer Immunol Res.

[19] Moya IM, Halder G (2019). Hippo-YAP/TAZ signalling in organ regeneration and regenerative medicine. Nat Rev Mol Cell Biol.

[20] Zhao H, Wang X, Yi P, Si Y, Tan P, He J (2017). KSRP specifies monocytic and granulocytic differentiation through regulating miR-129 biogenesis and RUNX1 expression. Nat Commun..

[21] Lemjabbar-Alaoui H, Hassan OU, Yang YW, Buchanan P (2015). Lung cancer: biology and treatment options. Biochim Biophys Acta.

[22] Byron E, Pinder-Schenck M (2014). Systemic and targeted therapies for early-stage lung cancer. Cancer Control..

[23] Kumar R, Collins D, Dolly S, McDonald F, O’Brien MER, Yap TA (2017). Targeting the PD-1/PD-L1 axis in non-small cell lung cancer. Curr Probl Cancer.

[24] Tian J, Rui K, Tang X, Ma J, Wang Y, Tian X (2015). MicroRNA-9 regulates the differentiation and function of myeloid-derived suppressor cells via targeting Runx1. J Immunol..

[25] Wu D, Shi M, Fan XD (2015). Mechanism of miR-21 via Wnt/beta-catenin signaling pathway in human A549 lung cancer cells and Lewis lung carcinoma in mice. Asian Pac J Trop Med..

[26] Lin L, Tu HB, Wu L, Liu M, Jiang GN (2016). MicroRNA-21 regulates non-small cell lung cancer cell invasion and chemo-sensitivity through SMAD7. Cell Physiol Biochem.

[27] Yang Y, Meng H, Peng Q, Yang X, Gan R, Zhao L (2015). Downregulation of microRNA-21 expression restrains non-small cell lung cancer cell proliferation and migration through upregulation of programmed cell death 4. Cancer Gene Ther.

[28] Li L, Zhang J, Diao W, Wang D, Wei Y, Zhang CY (2014). MicroRNA-155 and MicroRNA-21 promote the expansion of functional myeloid-derived suppressor cells. J Immunol..

[29] Chen Y, Zhang L, Liu L, Sun S, Zhao X, Wang Y (2018). Rasip1 is a RUNX1 target gene and promotes migration of NSCLC cells. Cancer Manag Res..

[30] Tian X, Ma J, Wang T, Tian J, Zheng Y, Peng R (2018). Long non-coding RNA RUNXOR accelerates MDSC-mediated immunosuppression in lung cancer. BMC Cancer..

[31] Wang Y, Dong Q, Zhang Q, Li Z, Wang E, Qiu X (2010). Overexpression of yes-associated protein contributes to progression and poor prognosis of non-small-cell lung cancer. Cancer Sci.

[32] Wang G, Lu X, Dey P, Deng P, Wu CC, Jiang S (2016). Targeting YAP-dependent MDSC infiltration impairs tumor progression. Cancer Discov.

[33] Zhang S, Liu Z, Wu L, Wang Y (2017). MiR-361 targets Yes-associated protein (YAP) mRNA to suppress cell proliferation in lung cancer. Biochem Biophys Res Commun.

[34] Han M, Wang F, Gu Y, Pei X, Guo G, Yu C (2016). MicroRNA-21 induces breast cancer cell invasion and migration by suppressing smad7 via EGF and TGF-beta pathways. Oncol Rep.

[35] Wang H, Xu W, Shao Q, Ding Q (2017). miR-21 silencing ameliorates experimental autoimmune encephalomyelitis by promoting the differentiation of IL-10-producing B cells. Oncotarget..

[36] Wang H, Fan H, Tao J, Shao Q, Ding Q (2019). MicroRNA-21 silencing prolongs islet allograft survival by inhibiting Th17 cells. Int Immunopharmacol.

